# Effect of bromocriptine on glycemic control, risk of cardiovascular diseases and weight in patients with type 2 diabetes: a systematic review

**DOI:** 10.1186/s13098-023-01073-2

**Published:** 2023-07-06

**Authors:** Mulualem Tesfaye Birhan, Teklie Mengie Ayele, Fikire Wondimu Abebe, Fiseha Nigussie Dgnew

**Affiliations:** 1 Department of Pharmacy, College of Health Science, Debre Tabor University, P.O.Box:272, Debre Tabor, 6300 Ethiopia; 2 Department of Pharmacology and Clinical Pharmacy, School of Pharmacy, College of health sciences, Addis Ababa University, Addis Ababa, Ethiopia

**Keywords:** Dopamine agonist, Diabetes mellitus, Bromocriptine, Glycemic control

## Abstract

**Background:**

Type 2 diabetes (T2DM) patients, including those in good glycemic control, have an increased risk of cardiovascular disease (CVD). Maintaining good glycemic control with drugs may reduce long-term CVD risk. Bromocriptine has been in clinical use for over 30 years, but the utility of bromocriptine in the treatment of diabetes patients has been proposed more recently.

**Objective:**

To summarize the available data regarding the effect of bromocriptine in T2DM management.

**Method:**

A systematic literature search was conducted in the electronic databases, including Google Scholar, PubMed, Medline, and Science Direct, to locate studies that meet the objectives of this systematic review. Additional articles were included by conducting direct Google searches of the references cited by eligible articles located by the database search. The following search terms were used on PubMed “bromocriptine OR dopamine agonist AND diabetes mellitus OR hyperglycemia OR obese”.

**Result:**

Eight studies were included in the final analysis. 6210 of the 9391 study participants received bromocriptine treatment, while 3183 received a placebo. The studies demonstrated that patients who took bromocriptine treatment had significantly reduced blood glucose and BMI, which is the main cardiovascular risk factor in T2DM patients.

**Conclusion:**

Based on this systematic review, bromocriptine may be used for T2DM treatment for its cardiovascular risk reduction effect, especially body weight reduction. However, advanced study designs might be warranted.

## Introduction

Type 2 diabetes (T2DM) is characterized by elevated fasting and postprandial plasma glucose concentrations, which result from increased endogenous glucose production (EGP), decreased insulin-mediated muscle glucose disposal, and suppression of endogenous glucose release, and inadequate pancreatic insulin secretion [1]. It is a growing global pandemic that is estimated to affect approximately 350 million people by the year 2030 [2].

The rise in prevalence of type T2DM in recent decades parallels the rise in obesity in both the United States and worldwide. It is estimated that one-third of individuals born in the past decade will develop T2DM [2]. The treatment cost of T2DM complication management may approach one-half of medical expenditures. The recommended targeted goals can prevent complications and improve outcomes, but these are only met by 50–70% of individuals with T2DM despite current therapies [3].

T2DM patients, including those in good glycemic control, have an increased risk of cardiovascular disease (CVD). Maintaining good glycemic control may reduce long-term CVD risk. However, other risk factors such as elevated vascular sympathetic tone and/or endothelial dysfunction may be stronger potentiators of CVD [4]. Various oral hypoglycemic agents have already been administered to type-2 diabetic patients to normalize their plasma glucose concentrations, but they have not had complete and sustained success [5]. As the underlying pathophysiology of T2DM is being unraveled, our pharmacological repertoire has expanded to target novel pathophysiological mechanisms. Even then, restoration of normoglycemia is difficult to achieve and requires multiple antidiabetic medications with different mechanisms of action, which can be used in combination to produce an additive effect [6]. The development of antidiabetic agents with novel mechanisms of action is therefore highly desirable [6].

However, despite the discovery of multiple antidiabetic agents, the effectiveness of glycemic control and reduction in cardiovascular risk are poor. Bromocriptine has been in clinical use for over 30 years, but the utility of bromocriptine in diabetes patients has been proposed more recently, based on its activity in modulating central glucose and energy metabolism pathways [2]. After several recent clinical trials, bromocriptine was approved by the United States Food and Drug Administration (FDA) for use in T2DM. The purpose of this paper is to review the current literature on the effects of bromocriptine in diabetes patients, including the glycemic control effect and cardiovascular risk reduction (especially weight reduction since obesity is the main risk factor for cardiovascular complications) effect of it.

## Methodology

A systematic review was conducted through database searching for literature and identified relevant studies that evaluate the effect of bromocriptine in T2DM management.

### Search strategy

A systematic literature search was conducted in the electronic databases of Google Scholar, PubMed, Medline, and Science Direct to locate studies that meet the objectives of this systematic review. Additional articles were included by conducting direct Google search of the references cited by eligible articles located by the database search. Electronic databases were searched using combinations of the following key terms ((bromocriptine) OR (Dopamine agonist))AND(Diabetes mellitus))OR(hyperglycemia))OR(Obese),(((“bromocriptine“[MeSH Terms] OR “bromocriptine“[All Fields] OR “bromocriptin“[All Fields] OR “bromocriptine s“[All Fields]OR(“dopamine agonists“[Pharmacological Action] OR “dopamine agonists“[MeSH Terms] OR (“dopamine“[All Fields] AND “agonists“[All Fields]) OR “dopamine agonists“[All Fields] OR (“dopamine“[All Fields] AND “agonist“[All Fields]) OR “dopamine agonist“[All Fields])) AND (“diabetes mellitus“[MeSH Terms] OR (“diabetes“[All Fields] AND “mellitus“[All Fields]) OR “diabetes mellitus“[All Fields])) OR (“hyperglycaemic“[All Fields] OR “hyperglycaemics“[All Fields] OR “hyperglycemic“[All Fields] OR “hyperglycemics“[All Fields])) AND (“obeses“[All Fields] OR “obesity“[MeSH Terms] OR “obesity“[All Fields] OR “obese“[AllFields]OR"obesities“[AllFields]OR"obesitys“[AllFields]))AND((y_10[Filter])AND(clinicaltrial[Filter]OR randomizedcontrolledtrial[Filter])AND(fft[Filter])). Key words like dopamine agonist, diabetes mellitus, bromocriptine, and glycemic control were important in searching on Medline database. A flow diagram was used to summarize the number of studies identified, screened, excluded, and finally included in the study.

### Inclusion and exclusion criteria

Only randomized control trials studies were included in the review. On the other hand, meta-analyses and other reviews, prospective studies, and retrospective studies, cohort studies, cross-sectional studies, and case reports were excluded. The PICO search strategy was used: “P” for T2DM patients), “I” for intervention (bromocriptine), “C” for comparison (placebo) and “O” for outcome (glycemic control, CV risk, or BMI reduction).

### Data extraction

Investigators extracted data by investigating the name of the first author and the year of publication, study area, study design, and sample size, which included the number of patients taking bromocriptine and placebo or other diabetes medications, sex, duration of follow up, effect of the trial on glycemic control, and patient weight. .

### Quality assessment

The JBI quality appraisal checklist for randomized controlled trials was used to evaluate each study’s level of quality [7]. The tool was used by three authors to evaluate each original study’s quality independently. Author disagreements that come up while critiquing the quality were resolved via discussions that were supported by facts. When there is any disagreement all the three authors discussed and resolved it. The critical appraisal checklist has thirteen parameters with yes, no, unclear and not applicable (NA) option. The parameter involves the following questions: (1) was true randomization used for assignment of participants to treatment groups? (2) Was allocation to treatment groups concealed? (3) Were treatment groups similar at the baseline? (4) Were participants blind to treatment assignment? (5) Were those delivering treatment blind to treatment assignment? (6) Were outcomes assessors blind to treatment assignment? (7) Were treatment groups treated identically other than the intervention of interest? (8) Was follow up complete and if not, were differences between groups in terms of their follow up adequately described and analyzed? (9) Were participants analyzed in the groups to which they were randomized? (10) Were outcomes measured in the same way for treatment groups? (11) Were outcomes measured in a reliable way? (12) Was appropriate statistical analysis used? (13) Was the trial design appropriate and any deviations from the standard RCT design (individual randomization, parallel groups) accounted for in the conduct and analysis of the trial? If a study received a score of at least 50% on each of the quality assessment criteria for the study design, it was deemed low risk.

## Result

**Fig. 1:**
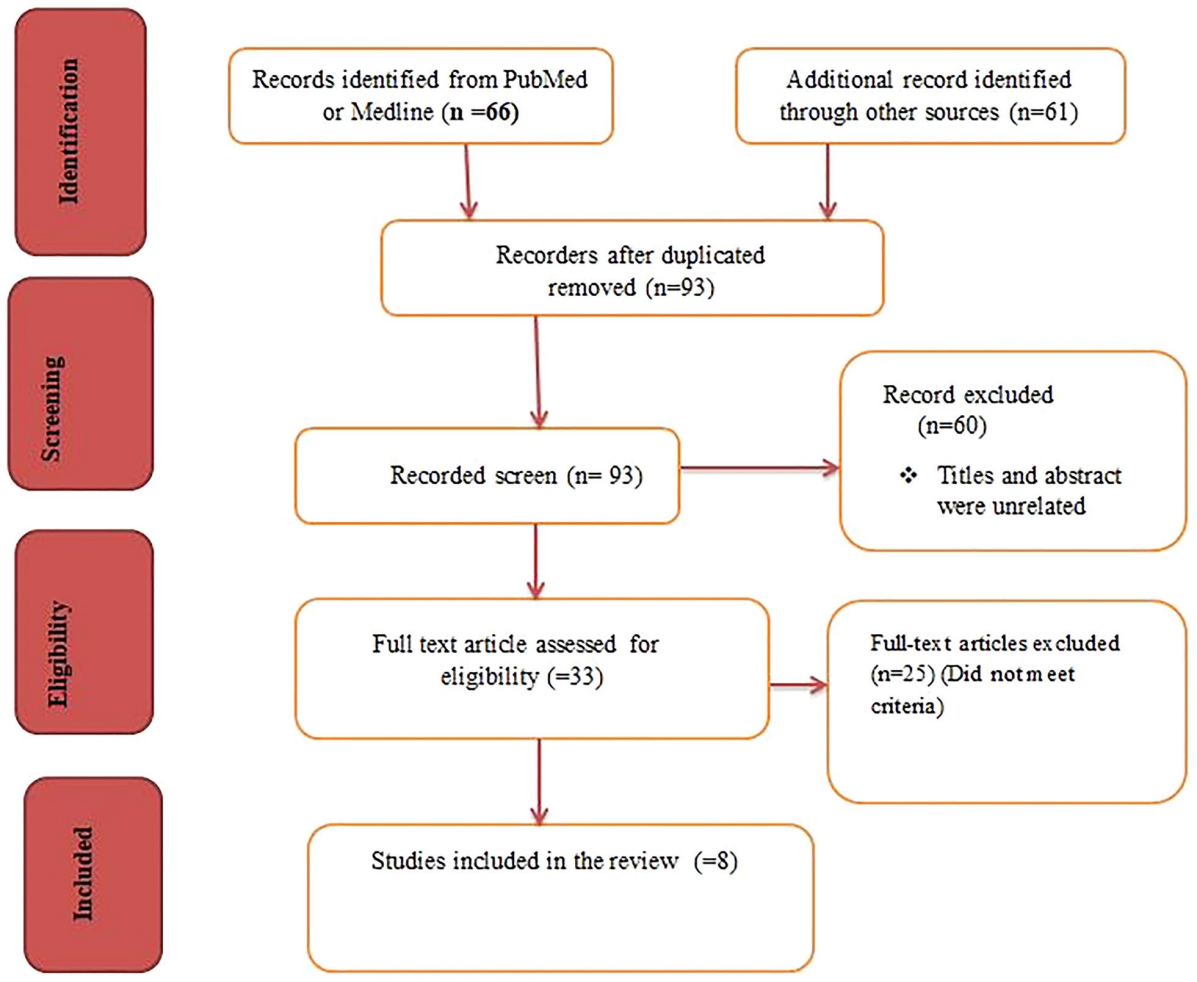
Shows the flow Figure of screened excluded and included studies

A total of 127 studies were identified through the initial electronic database search (49 via PubMed, 60 via Google Scholar, 6 via Medline, and 5 via science Direct). Additional 7 articles were identified through reference searching. 34 articles were removed due to duplicates. Then 93 articles were screened, and 60 articles were removed due to unrelated topics and interest in the outcome. Furthermore, based on the eligibility criteria, 33 full articles were reviewed, and 25 articles that did not meet the inclusion criteria were removed. Finally, for the final review, 8 full articles (randomized controlled trials) were included. The figure below briefly describes the flow of the study selection employed (Fig. 1). Additionally, the authors evaluated the all randomized controlled trials for the quality of methodology using JBI Quality Assessment tool (Table 1). A positive response (yes) indicates higher quality methodology.

**Table 1:** Quality assessment of randomized clinical trials with JBA tool

Studies	Answers			
	Yes	No	Unclear	NA
Aminorroaya et al. [5]	*			
Chamarthi et al. [8]	*			
Chamarthi et al. [9]	*			
Cincotta et al. [12]	*			
Chamarthi and Cincotta [16]	*			
Pijl et al. [1]	*			
Gaziano et al. [11]	*			
Gaziano et al. [2]	*			

Ratings scales are based on the parameters: (1) was true randomization used for assignment of participants to treatment groups? (2) Was allocation to treatment groups concealed? (3) Were treatment groups similar at the baseline? (4) Were participants blind to treatment assignment? (5) Were those delivering treatment blind to treatment assignment? (6) Were outcomes assessors blind to treatment assignment? (7) Were treatment groups treated identically other than the intervention of interest? (8) Was follow up complete and if not, were differences between groups in terms of their follow up adequately described and analyzed? (9) Were participants analyzed in the groups to which they were randomized? (10) Were outcomes measured in the same way for treatment groups? (11) Were outcomes measured in a reliable way? (12) Was appropriate statistical analysis used? (13) Was the trial design appropriate and any deviations from the standard RCT design (individual randomization, parallel groups) accounted for in the conduct and analysis of the trial?

### Characteristics of included studies

A total of 8 studies (9391 study participants, 59.6% of whom were male) were included in this review. All off the included studies were randomized controlled trials (1, 2, 5, 8–12). Most of the studies were conducted in the USA in multicenter settings; only one study was conducted in Asia [5]. 66.12% of the participants were on bromocriptine, and the remaining 33.9% were on a placebo (Table 2).

**Table 2:** Characteristics of included studies

Author, year	Study area	Study design	Sample size	Sex		Participants on		Bromocriptine effect			P value, OR of HR			Duration of follow up
				M	F	Bromocriptine	Placebo							
								HbA1	Fasting glucose	BMI Or CV risk	HbA1	FPG	BMI Or CV risk	
Aminorroaya et al. [5]	Asia (Iran)	RCT	40	6	34	20	20	Reduced compared with the placebo	Reduced	No change	p < 0.01	P < 0.05		52wks
Chamarthi et al. [8]	USA (Boston)	RCT	1834	1008	826	1219	615	Reduced	Reduced	Reduced	p=0.002	p=0.002	HR: 0.52 [0.28 − 0.98	18wks
Bindu et al. [9]	Boston	RCT	1791	1025	765	1208	583	Reduced	Reduced	Reduced	P < 0.003	P < 0.001	HR: 0.45(p = 0.028	52wks
Cincotta et al. [12]	Charlestown and massachusetts	RCT	17	10	7	10	7	Reduced	Reduced	Reduced	P < 0.02	P < 0.02	P < 0.01	18wks
Chamarthi and Cincotta [16]	USA (Bonton)	RCT	60	50	10	44	16	Reduced	No significant difference	NA	P < 0.001	P = 0.49	NA	12wks
Pijl et al. [1]	San Antonio	RCT	22	8	14	15	7	Reduced	Reduced	No change	P = 0.009	P < 0.02	P > 0.13	16wks
Gaziano et al. [11]	USA(multicenter)	RCT	3070	1739	1331	2054	1016	Reduce	Reduced	Significant reduction	P = 0.12	0.12	52%reduction in relative risk(p < 0.05)	52wks
Gaziano et al. [2]	Charlestown and massachusetts	RCT	2557	1739	818	1643	914	Reduced	Reduced	Reduced	P < 0.05	P < 0.05	P < 0.001	24wks

Among the included studies, the majority of them [2, 8–11] showed the benefit of bromocriptine in T2DM cardiovascular risk reduction, especially BMI reduction, which is the main cardiovascular risk factor in T2DM patients. Almost all the studies showed the ability of this drug to improve glycemic control among T2DM patients. Only two of the studies [1, 5] showed that there was no difference in body fat or body weight reduction between treatment and control groups.

### Glycemic control effect of bromocriptine

From the study included in almost all of them [1, 2, 5, 8–11], showed bromocriptine significantly reduced HbA1 (Hemoglobin A1C) and FPG (Fasting Plasma Glucose) in T2DM patients. For example, a double blind randomized control study by Aminorroaya et al. [5] showed a decline in both HbA1 and FPG in the bromocriptine treatment group, while there was no change in the placebo group. The FPG level decreased in the bromocriptine-treated group from 10.59 ± 0.42 to 9.06 ± 0.41 mmol/l (p = 0.01) and the HbA1 concentration was reduced in the bromocriptine-treated group from 9.9 ± 0.3 to 9.5 ± 0.2% (p = 0.06), whereas in the placebo group the FPG level was unchanged at10.69 ± 0.52 and 10.6 ± 0.57 mmol/l, respectively, but the HbA1c level was increased from 10.2 ± 0.3 to 11.3 ± 0.6%. A double blind randomized control study [1] showed that bromocriptine significantly reduced HbA1c (from 8.7 to 8.1%, P = 0.009) and fasting plasma glucose (from 190 to 172 mg/dl, P = 0.02), whereas these variables increased during placebo treatment. Among the included studies, the study by Bindu and Anthon (2017) showed different results which showed no significant change in FPG level in the bromocriptine treatment group compared with the placebo group but a significantly reduced HbA1 level in the bromocriptine treatment group compared with the placebo group. A randomized clinical trial of quick-release (QR) bromocriptine among patients with T2DM on overall safety and cardiovascular outcomes [2] stated the glycemic control effect of bromocriptine as a cardiovascular risk reduction effect associated with control of hyperglycemia and reduction in body fat, so bromocriptine could reduce hyperglycemia.

### Cardiovascular risk reduction

Majority of the included studies showed that bromocriptine in T2DM reduces cardiovascular risk by reducing body weight, lipid level, or direct control of glycemic [2, 8, 9, 11, 12]. A randomized control study conducted by Gaziano et al. [11] found 14 events (0.7%) among 2054 bromocriptine-QR-treated subjects and 15 events (1.5%) among 1016 placebo-treated subjects, yielding a significant, 52% reduction in relative risk in this end point with bromocriptine-QR exposure (P˂0.05; log-rank test) regarding the major adverse cardiovascular events end point, and this relative risk reduction was consistent regardless of age, disease duration, race, sex, or preexist [11]. Gaziano et al. [2] conducted a similar RCT in T2DM patients and found that bromocriptine reduced cardiovascular risk by 40% (HR 0.60 [95% two-sided CI 0.37–0.96]) compared with placebo, regardless of age, disease duration, race, sex, or preexisting CV disease (2). A double-blind, placebo-controlled study investigating the effects of a new quick release formulation of bromocriptine on body weight, body fat, and glucose tolerance in a group (n = 17) of obese subjects who were instructed to follow a moderate hypocaloric diet showed that bromocriptine treatment for 18 weeks significantly reduced body weight and body fat versus placebo (6.3 ± 1.5 and 5.4 ± 1.1 kg vs. 0.9 ± 1.0 and 1.5 ± 0.6 kg, respectively, P < 0.01). This effect directly reduces cardiovascular risk since those risks are associated with a high BMI and hyperlipidemia [12].

However, some of the included studies found no significant difference in BMI or fat reduction effect of bromocriptine compared to placebo [1, 5]. A double-blind, placebo-controlled, randomized study in 40 obese patients with T2DM showed that, although there were significant reductions in FPG and HbA1c levels in the bromocriptine treatment group compared with the placebo group, no changes in body weight or BMI occurred during the study in either the placebo- or bromocriptine-treated group’s BMI during the entire study period (bromocriptine, 33.2 ± 1.2 vs. 33.2 ± 1.2 kg/m^2^; placebo, 31.8 ± 1.0 vs. Similarly, a 16-week double-blind study on 22 obese subjects with T2DM randomized to receive a quick-release formulation of bromocriptine (n = 15) or a placebo (n = 7) showed the result that no changes in body weight or body composition occurred during the study in either placebo- or bromocriptine-treated subjects, rather that body weight was associated with significantly reduced FPG and HbA1c levels [1].

## Discussion

According to the above studies, which demonstrate metabolic improvements after the administration of bromocriptine, including significant weight loss, decreased levels of blood glucose and triglycerides, and decreased insulin resistance. These indicate that bromocriptine has a glycemic control and cardiovascular risk reduction effect on T2DM treatment.

A Systematic Review and Meta-Analysis on Efficacy and Safety of bromocriptine-QR in T2DM conducted by Liang et al. [2015] supports the result by half; the review supports the glycemic control effect of bromocriptine but opposes the CV risk reduction effect as bromocriptine-QR exhibited an increase in achieving an HbA1c level of 7.0% (32.0 vs. 9.5%; odds ratio, 4.57; 95% CI 2.42–8.62) [13]. FPG was reduced with bromocriptine-QR compared with placebo (weighted mean difference, 1.04 mmol/l; 95% CI 1.49–0.59 mmol/l); however, bromocriptine-QR had neutral effects on postprandial glycemia, BMI, and lipid profile [13].

Expert Review of Endocrinology & Metabolism on bromocriptine effect in T2DM supports the result as across studies of various T2DM populations, bromocriptine-QR has been demonstrated to reduce HbA1c by −0.5 to −1.7 and associated with a 42% hazard ratio reduction of a pre-specified adverse cardiovascular endpoint, including myocardial infarction, stroke, hospitalization for congestive heart failure, and revascularization surgery, but no identified effect on FPG level [14]. Similarly, the Journal of Diabetes Investigation case report supports the result of this review [15].

The glycemic control effect of bromocriptine is associated with a reduction in FPG and HbA1c levels, which is supported by most of the included studies, but Chamarthia and Cincotta (2017) showed the effect occurred without change in FPG which suggests a postprandial glucose lowering mechanism of action (reduction in HbA1c). After 12 weeks of bromocriptine—QR treatment, mean % HbA1c decreased by −0.73% relative to baseline (p < 0.001) and by −1.13 relative to placebo (p < 0.001), whereas the FPG change within each treatment group was not significant [16]. Bromocriptine was able to control glycaemia in poorly controlled DM on metformin plus basal-bolus insulin, including individuals on high-dose basal-bolus insulin.

A systematic review conducted by Michael et al. [2010] demonstrated that, in addition to the glycemic control and CV risk reduction benefits of bromocriptine, once-daily schedule is important for adherence [3]. Diabetes patients often cite factors such as medication cost, forgetfulness to take medications, comorbid depression, and concern for weight gain, hypoglycemia or other adverse effects as reasons for not adhering to prescribed medication regimens. With regard to these factors, bromocriptine has advantages over other new and existing diabetes therapies. For patients who do not always remember to take their medications, the once-daily dosing schedule of bromocriptine fits easily into most daily routines, which may aid in medication adherence [3]. But in this review, this effect not identified.

## Conclusion

Glycemic controls along with cardiovascular protection are concerning issues in diabetic patients. Most of the antidiabetic drugs have weight gain as a side effect that ends in cardiovascular complications. Based on this systematic review, bromocriptine may be used for T2DM treatment for its cardiovascular risk reduction effect, especially body weight reduction. However, further study might be warranted.FV

## Data Availability

All the data sets generated and analyzed during the study are included in the text.

## Abbreviations

BMI: Body mass index
CVD: Cardiovascular disease
FPG: Fasting plasma glucose
FDA: Food and drug administration
HbA1C: Hemoglobin A1C
HR: Hazard ratio
NA: Not assessed
PRISMA: Preferred reporting items for systematic reviews and meta analyses
RR-B: Rapid released bromocriptine
T2DM: Type 2 diabetes mellitus
QR-B: Quick released bromocriptine
